# Aqueous leaf extract of *Clinacanthus nutans *improved metabolic indices and sorbitol‐related complications in type II diabetic rats (T2D)

**DOI:** 10.1002/fsn3.988

**Published:** 2019-03-07

**Authors:** Mustapha Umar Imam, Maznah Ismail, Annie George, Sasikala M. Chinnappan, Ashril Yusof

**Affiliations:** ^1^ Laboratory of Molecular Biomedicine, Institute of Bioscience University of Putra Malaysia Selangor Malaysia; ^2^ Department of Nutrition and Dietetics, Faculty of Medicine and Health Sciences University of Putra Malaysia Selangor Malaysia; ^3^ Faculty of Science, Institute of Biological Sciences University of Malaya Kuala Lumpur Malaysia; ^4^ Biotropics Malaysia Berhad Shah Alam Malaysia; ^5^ Exercise Science, Sports Centre University of Malaya Kuala Lumpur Malaysia

**Keywords:** Aldose reductase, antidiabetic, antioxidant activity, sorbitol dehydrogenase

## Abstract

*Clinacanthus nutans *(Burm. f.) Lindau (*C. nutans*) has been reported to lower blood glucose level; however, evidence on its efficacy in lowering diabetic complications is limited. The antidiabetic properties of *C. nutans *aqueous leaf extract on serum metabolic indices, sorbitol production, and aldose reductase enzyme activities in the kidneys, lens, and sciatic nerve of type II diabetic (T2D) rats were evaluated. All rats except normal control rats were fed with a high‐fat diet for 8 weeks to induce obesity and subsequently injected with 35 mg/kg streptozotocin to induce type II diabetes. Aqueous leaf extract of *C. nutans* (100 and 200 mg kg^−1^ day^−1^) and quercetin (10 mg kg^−1^ day^−1^) were fed orally for 4 weeks. Diabetic rats administered with *C. nutans *at 100, 200 mg kg^−1^ day^−1^ and quercetin had significantly (*p < *0.05) lower fasting blood glucose levels post‐intervention: 14.2, 14.0, and 19.9 mm, respectively, compared with the untreated group (22.1 mm). Total cholesterol was significantly (*p* < 0.05) lower in the *C. nutans* groups in comparison with the diabetic control group. Levels of F2‐isoprostane, a marker of oxidative stress, were attenuated in the presence of the extract. Aldose reductase enzyme activity increased by 64, 99, and 0% and total antioxidant activities by 22, 29, and 126%, respectively. Sorbitol levels in the kidney, lens, and nerve were reduced in diabetic rats administered with *C. nutans *and quercetin group (by 8, 16, and 3%, respectively). The protective effect of the extract to the liver and kidney was confirmed through liver and kidney enzyme markers and histological analyses. The *C. nutans* has the potential to attenuate T2D‐induced metabolic perturbations and complications related to sorbitol accumulation.

## 1 INTRODUCTION

Type II diabetes (T2D) is currently reaching pandemic proportions, and despite advances in managing this disease, the incidence and prevalence keep rising. The perceptions of T2D are changing, as well as its management. Lifestyle factors involving diet now feature prominently as the more important contributors to the pathogenesis of T2D than genetics (Chopra, Galbraith, & Darnton‐Hill, 2002; Hu, 2011). Plants have been traditionally used to manage communicable and noncommunicable diseases, especially in Africa and Asia. The realization that medicinal plants provide a cheaper means of disease management has generated interest in their use globally, and has driven the search for newer therapies. Many of these plants have been used solely based on a traditional hunch, and studies are now providing evidence of their efficacy. Therefore, there is an increasing drive to study natural products in the form of edible plants or herbs which can be used as part of the daily diet to manage T2D (Gurib‐Fakim, 2006). The effectiveness of the management of T2D depends largely on the ability of the therapeutic agent to control complications as chronic hyperglycemia is associated with long‐term damage and failure of various organs such as eyes, nerves, kidneys, and the heart (Chawla, Chawla, & Jaggi, 2016). An antioxidant‐rich supplementation in T2D patients can assist in debilitating complications of diabetes by increasing the total antioxidant levels and reducing oxidative stress (Ganjifrockwala, Joseph, & George, 2017). Hence, those plants that show potential for affecting not only antihyperglycemia but also antioxidation and the control of other complications of diabetes are good candidates for managing the diabetes mellitus.

The burden of T2D is increasing in developing countries more than in developed countries (World Health Organisation, 2014). Potentially, a novel candidate plant is *C. nutans *which is traditionally used for the management of numerous medical conditions (National Drug Committee, 2006), while studies have indicated that its extracts have blood glucose‐lowering (Nurulita, Dhanutirto, & Soemardji, 2008), antioxidant (Pannangpetch et al., 2007), anticancer (Yong, Tan, & Tehetal, 2013), and anti‐inflammatory effects (Wanikiat et al., 2008). Interestingly, it is categorized as an essential medicinal plant for primary health care by the Thai Ministry of Public Health (National Drug Committee, 2006). Sadly, extensive investigations into the antidiabetic properties of *C. nutans* including its potential to prevent diabetic complications have not been performed. Moreover, with the growing interest in plant bioresources as potentially cost‐effective and safer alternatives to available drugs for the management of T2D, it is hoped that in‐depth functional analyses of *C. nutans* may pave the way for its use in the management of metabolic disorders. Thus, in the present study, we evaluated the effects of *C. nutans* on cardiometabolic indices and sorbitol‐related complications in a T2D rat model, in order to provide insight into the basis for its functional effects, which will encourage its evidence‐based usage.

## 2 MATERIALS AND METHODS

### 2.1 Materials

All chemicals including quercetin, a well‐known flavonoid antioxidant known to protect against oxidative stress in T2D rat model (Yang & Kang, 2018), were purchased from either Sigma‐Aldrich Chemical (USA) or Thermo Fisher Scientific (MA, USA). Enzyme‐linked immunosorbent assay (ELISA) kits (F2‐isoprostanes, aldose reductase, sorbitol dehydrogenase, and insulin) were purchased from Elabscience Biotechnology Co. Ltd (Wuhan, China), while the total sorbitol content kit was purchased from Sigma Chemical Co. (St. Louis, MO, USA). The deproteinization kit was purchased from Biovision (Mountain View, CA, USA), and RCL2® Solution was purchased from ALPHELYS (Toulouse, France). The fine sugar and starch powders used to make pellets were purchased from R & S Marketing Sdn. Bhd. (Malaysia), and the palm oil, Nespray fortified milk powder, and standard rat chow were purchased from Unilever (Malaysia), Nestle Manufacturing (Malaysia), and Specialty Feeds (TN, USA), respectively.

#### 2.1.1 Plant materials

An amount of 3 kg *C. nutans *leaves were purchased from RD Agro Herbs, Serdang, Malaysia. Authentication was performed at Forest Research Institute Malaysia (FRIM) where the voucher specimen PID 300615‐16 was deposited. The collected leaves were washed thoroughly with running tap water and repeatedly with deionized water. The aerial parts, including leaves and stems, were air‐dried for 4 days prior to further analyses.

#### 2.1.2 Hot water extraction

The leaves of *C. nutans *were cut into 5 mm pieces in a cutting mill and passed through the mesh of a 6.0 mm sieve (Retsch SM 100, Germany). The leaves were then mixed with water in a 1:10 ratio (w/v) and heated using a hot plate for 3 hr at 100°C. The mixtures were subsequently filtered through cotton wool, and the water was removed under reduced pressure (Rotavapor R210, Buchi, Switzerland) followed by spray drying (Buchi mini spray drier B‐290, Switzerland). The dried crude extracts were stored at room temperature until further analyses.

### 2.2 Aldose reductase activity of plant extract

The extracts (10–100 µg/ml) and quercetin (0.05–1.5µM) were dissolved in DMSO and their aldose reductase activities were evaluated using the aldose reductase ELISA kit (Yagihashi et al., 2001). Absorbance (OD) was recorded at 340 nm, and the percentage of inhibitory activity was calculated according to Hsu (2006) (Equation 1):

(1) $$\text{Percentage}\;\text{of}\;\text{inhibitory}\;\text{activity}=\left[\frac{\left(\text{absorbance}\;\text{of}\;\text{control}-\text{absorbance}\;\text{of}\;\text{sample}\right)}{\text{absorbance}\;\text{of}\;\text{control}}\right]\times 100$$

### 2.3 Animal handling

Male Sprague Dawley rats (10 weeks old, weighing 200‐250g, *n* = 25) were obtained from the Department of Companion Animal Medicine & Surgery, Veterinary Medicine Faculty, University of Putra Malaysia. The rats were housed in pairs in plastic cages and allowed normal rat chow and water ad libitum for 2 weeks to acclimatize animals under controlled conditions (25–30°C, with a 12/12 hr light/dark cycle). Animal ethics approval was given by the Animal Care and Use Committee (ACUC) of the Faculty of Medicine and Health Sciences, University Putra Malaysia (Project approval number: UPM/IACUC/UAP‐R045/2013), and the guidelines for the use of animals were strictly adhered to. After acclimatization, five rats were maintained on normal rat chow, while the rest were fed high‐fat diet (47.7% total carbohydrate, 16.1% protein, 31.1% fat, 2.5% fiber, and 5.1% mineral and vitamin mix) for 8 weeks to induce obesity. Following the induction of obesity, streptozotocin (STZ) was injected intraperitoneally (35 mg/kg in sodium citrate buffer, pH 4.5) to induce T2D (Imam & Maznah, 2013). Normal control rats were given 5 mmol/L of sodium citrate buffer, pH 4.5 intraperitoneally. The newly diabetic rats (fasting plasma glucose of ≥ 250 mg/dl after 2 days of STZ) were divided into four groups (*n* = 5): diabetic control, 100 mg kg^−1^ day^−1^ aqueous leaf extract of *C. nutans*, 200 mg kg^−1^ day^−1^ aqueous leaf extract of *C. nutans,* and quercetin (10 mg kg^−1^ day^−1^) groups. Food intake was maintained at 30 kcal/100 g body weight/day, which lasted for another 4 weeks together with oral feeding of quercetin and extracts. The animals were then euthanized using diethyl ether and subsequently exsanguinated, and their blood and organs (liver, kidney, lens, and sciatic nerves) harvested for serum biochemical analyses and tissue histology, total antioxidant, sorbitol content, aldose reductase, and sorbitol dehydrogenase testing. Weekly measurements of weight and fasting blood glucose were made using a laboratory measuring scale and glucometer (Accu‐Chek Performa), respectively.

### 2.4 Harvesting of blood, liver, lens, kidney, and sciatic nerve

The organs were obtained from the experimental rats, immediately washed with ice‐cold saline, dried with filtered paper, and then stored in RCL2® Solution (ALPHELYS, France) at −80°C. Blood was collected by cardiac puncture after an overnight fast, and about 10 ml of blood was drawn upon exsanguinations from each rat and was centrifuged at 978 *g* for 10 min at 4°C to separate the serum. Additionally, serum from blood collected at the beginning of the experiment (after the induction of T2D) and at the end of the experiment was used to evaluate changes in biochemical indices (serum lipid profile concentrations, liver enzymes activities, serum creatinine, and urea) concentrations induced by feeding the extracts. For assays requiring tissue samples, the frozen tissues were thawed and homogenized immediately prior to each assay.

### 2.5 Detection of serum insulin, liver antioxidant status, and F2‐isoprostane

For these assays, 100 mg of liver samples was homogenized in 1 ml cold phosphate‐buffered saline (1× PBS, pH 7.4), centrifuged at  5323 *g* for 5 min, and the supernatant was collected. Serum insulin, liver F2‐isoprostane, and total antioxidant status were quantified using ELISA kits (Elabscience Biotechnology Co. Ltd, Wuhan, China) according to the manufacturer's instructions. The absorbance was read at 450 nm on a BioTeK Synergy H1 Hybrid Reader (BioTek Instruments Inc., Winooski, VT, USA).

### 2.6 Kidney, lens, and sciatic nerve antioxidant status, total sorbitol, sorbitol dehydrogenase and aldose reductase

Tissues stored in RCL2 (a new non cross‐linking fixative) solution (kidney, lens, and nerve) were homogenized in cold phosphate‐buffered saline (1× PBS, pH 7.4), centrifuged at 5323 *g* for 5 min, and the supernatant used for total antioxidant status and activities of aldose reductase and sorbitol dehydrogenase. For sorbitol quantification, samples were deproteinized according to the kit protocol (Deproteinization kit, Biovision, Mountain View, CA, USA) prior to the assay. Tests were conducted using supernatants from the kidney, lens, and nerves using the respective kits according to the manufacturer's instructions.

### 2.7 Histological analyses

To analyze safety of use of *C. nutans*, the effect of the extract was investigated further by the histological sections of the liver and kidney. A 2 cm × 2 cm sections of each organ were fixed in 4% paraformaldehyde overnight, embedded in paraffin, and cut into thin sections. The sections were stained with hematoxylin and eosin (H&E) and analyzed using a light microscope (Nikon ECLIPSE TS100, Nikon Corporation, Tokyo, Japan) attached to a digital camera (Nikon DS‐Fi1, Nikon Corporation, Tokyo, Japan) with image acquisition software (NIS Elements D 3.0 version).

### 2.8 Data analysis

Data are presented as means ± *SD* (*n* = 5). Data were checked for homogeneity using the Shapiro–Wilk test and *p* > 0.05. Two‐way repeated analysis of variance (ANOVA) of group versus time using SPSS 21.0 software (SPSS Inc., Chicago, IL, USA) to assess differences in means for blood glucose and body weight was employed while one‐way ANOVA was used to analyze differences in means for other parameters measured. Different factors/parameters needed to be assessed taking into consideration how they fulfill the criteria for statistical analyses. For the one‐way ANOVA, it tests differences between three or more means, while the two‐way ANOVA assesses the interrelationship of two independent variables on a dependent variable. In this case, it was more convenient to use different methods for ease of analysis. Statistical significant was set at *p* < 0.05. For insulin, antioxidant and F2‐isoprostane, the results were analyzed at www.myassays.com using linear regression equation (insulin, *R* ^2^ = 0.9954; *R* ^2^ = 0.984; antioxidant status, *R* ^2^ = 0.94) and four parametric test curve (F2‐isoprostane).

## 3 RESULTS AND DISCUSSION

### 3.1 Aldose reductase activity of plant extract

**Figure 1:**
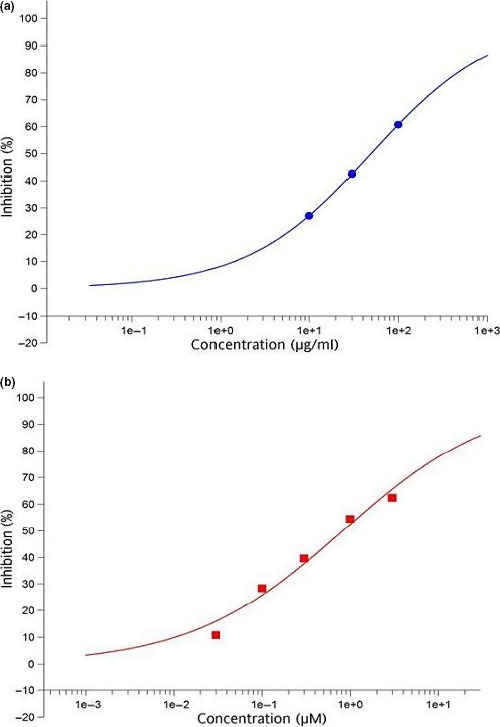
Inhibitory effects of *Clinacanthus nutans* (a) and quercetin (b) on aldose reductase in vitro

The effect of *C. nutans *aqueous leaf extract on aldose reductase is shown in Figure 1. At 10 µg/ml, there was only 27% inhibition of activity, while at 100 µg/ml, 61% of the enzyme activity was inhibited, with an IC_50_ of 49.4 µg/ml. Quercetin, on the other hand, showed a potent aldose reductase inhibitory effect, with an IC_50_ of 0.82 µM (2.7 µg/ml). Both quercetin and aqueous leaf extract of *C. nutans* had aldose reductase inhibitory effects. The data suggest that although quercetin had potent aldose reductase inhibitory effects, in line with its known effects (Patel, Kumar, Kumar, Sairam, & Hemalatha, 2012), aqueous leaf extract of *C. nutans* was also a candidate for such effects, especially in view of the continuing search for safer and more cost‐effective alternative therapies for the management of diabetic complications (Gurib‐Fakim, 2006).

### 3.2 Blood glucose, serum insulin, and body weight

Table 1 shows glucose and weight changes over 4 weeks of intervention. For blood glucose, there was a significant interaction between group and time (*F* = 2.16, *p = *0.011). There was a significant time effect (*F* = 6.25, *p* = 0.0002) where a progressive increase in glycemia in the diabetic control group was observed while the *C. nutans *and quercetin groups showed improved glycemic control. Interestingly, both concentrations of *C. nutans* showed to be more effective than quercetin (group effect; *F* = 110.41, *p < *0.0001). There were no differences in serum insulin levels between the diabetic treatment groups and untreated (Table 2) suggesting that the differences observed in glycemia may not have been due to effects of the extracts on insulin levels but likely due to enhanced activity of insulin or an insulin‐like action. Meanwhile, the changes in weight were not considered significant between treated and untreated rats at all time points. However, the percentage change (normalized to baseline) showed significant differences between *C. nutans* (200 mg/kg) and quercetin (5 and 8% respectively) with untreated group (17%) with *p < *0.05.

**Table 1:** Blood glucose and body weight changes over 4 weeks of intervention

Rat groups	Baseline	Week 1	Week 2	Week 3	Week 4
Glucose (mM)					
*C. nutans* 100 mg/kg	18.6 ± 0.7^a^	14.6 ± 2.8^a^^,^ b	14.0 ± 2.2^a^	14.2 ± 2.1^a^	14.2 ± 2.1^a^
*C. nutans* 200 mg/kg	19.5 ± 0.81^a^	14.5 ± 2.1^a^^,^ b	15.4 ± 4.2^a^	15.0 ± 2.1^a^	14.1 ± 4.2^a^
Quercetin 10 mg/kg	19.8 ± 4.1^a^	14.8 ± 3.4^a^	16.8 ± 1.2^a^	20.0 ± 4.3^a^	19.9 ± 4.2^a^
Diabetic control	18.2 ± 0.8^a^	15.2 ± 1.8^a^	17.7 ± 2.3^a^	18.3 ± 1.6^a^	22.1 ± 4.8^a^
Normal	4.7 ± 0.5	4.2 ± 0.2	5.3 ± 1.3	5.3 ± 0.9	5.2 ± 0.8
Weight (g)					
*C. nutans* 100 mg/kg	326 ± 30^a^	293 ± 40^a^	282 ± 44^a^	283 ± 45^a^	279 ± 44^a^^,^ b
*C. nutans* 200 mg/kg	306 ± 30^a^	273 ± 29^a^	287 ± 21^a^	291 ± 30^a^	290 ± 40^a^^,^ b
Quercetin 10 mg/kg	322 ± 57	302 ± 44	297 ± 53	303 ± 52	296 ± 48
Diabetic control	311 ± 21	278 ± 29	261 ± 39	261 ± 38	258 ± 38^a^
Normal	270 ± 21	288 ± 24	303 ± 25	316 ± 25	325 ± 26

Note

Data are mean ± *SD* (*n* = 5). The normal group was fed normal pellet throughout the intervention, while the diabetic control group (high‐fat diet + 35 mg/kg streptozotocin) received high‐fat diet for 4 weeks. The quercetin, *C. nutans *100 mg and *C. nutans* 200 mg groups received quercetin (10 mg kg^−1^ day^−1^), and 100 mg kg^−1^ day^−1^ and 200 mg kg^−1^ day^−1^ of aqueous extract of *Clinacanthus nutans* in addition to the high‐fat diet for 4 weeks after diabetes induction.

a Statistical difference in comparison with the normal group for either glucose or weight measurements in each column (*p* < 0.05).

b Statistical difference in comparison with the diabetic control normal group in each row (*p* < 0.05).

**Table 2:** Serum biochemical parameters in type 2 diabetic rats at 4 weeks of intervention

Parameter	*C. nutans *(100 mg/kg)	*C. nutans* (200 mg/kg)	Quercetin (10 mg kg^−1^ day^−1^)	Diabetic control	Normal
Insulin (pg/ml)	157 ± 13^a^	150 ± 17^a^	140 ± 10^a^	125 ± 32^a^	265 ± 24
TC (mM)	1.3 ± 0.24^b^	1.2 ± 0.18^b^	1.3 ± 0.11^b^	1.7 ± 0.03^a^	1.2 ± 0.12
Trig (mM)	0.8 ± 0.34^b^	0.6 ± 0.11^b^	1.2 ± 0.08^a^^,^ b	1.5 ± 0.04^a^	0.6 ± 0.11
LDL (mM)	0.2 ± 0.05^b^	0.2 ± 0.07^b^	0.3 ± 0.07^b^	0.4 ± 0.02^a^	0.2 ± 0.06
HDL (mM)	0.9 ± 0.15^b^	1.0 ± 0.13^b^	0.8 ± 0.16	0.7 ± 0.09^a^	0.9 ± 0.05
ALT (U/L)	59.5 ± 12.40	60 ± 13.19	73 ± 19.8	90.1 ± 36.39	57.6 ± 12.46
ALP (U/L)	229.3 ± 63.53^a^^,^ b	211 ± 72.43^a^^,^ b	454 ± 11.55^a^	440.3 ± 33.68^a^	126.3 ± 4.74
AST (U/L)	109.1 ± 15.40	123.2 ± 16.16	148.3 ± 60.44	188.1 ± 50.9	149.9 ± 31.12
Creatinine (μM)	62.0 ± 7.19	64.0 ± 5.15	67 ± 7.75	72.5 ± 10.76	60.5 ± 3.51
Urea (mM)	5.3 ± 1.42	5.3 ± 1.56	5.5 ± 1.23	6.8 ± 0.56^a^	4.2 ± 0.88

Note

Data are mean ± *SD* (*n* = 5). Groups are the same as in Table 1.

ALT: alanine transaminase; AST: aspartate transaminase; Crea: creatinine; HDL: high density lipoprotein; LDL: low density lipoprotein; TC: total cholesterol; Trig: triglyceride.

a Statistical difference in comparison with the normal in each row (*p* < 0.05).

b Statistical difference in comparison with the diabetic control normal group in each row (*p* < 0.05).

Sustained chronic hyperglycemia is the hallmark of T2D (Stumvoll, Goldstein, & Van Haeften, 2005). The improved glycemic control due to *C. nutans *leaf aqueous extract in comparison with quercetin suggested that aqueous leaf extract of *C. nutans* could produce better glycemic outcomes in T2D when compared to quercetin. Moreover, improvements in glycemic control have been linked with improved overall control of T2D (Landman, 2010).

The weight of the diabetic control group though not statistically significant decreased progressively during the intervention period (Table 1), suggesting that gluconeogenesis was taking place (Imam & Maznah, 2013). Gluconeogenesis promotes the breakdown of fats and proteins in T2D for conversion to glucose, which can be attenuated by quercetin (Gasparin, Spitzner, Ishii‐Iwamoto, Bracht, & Constantin, 2003). Interestingly, the 17% weight loss in the diabetic control group between baseline and end of study was significantly difference to the quercetin group (8% of their weight). The weight loss for the *C. nutans* groups was 15 and 5% for the low and low dose extracts, respectively. The results from the study showed that the *C. nutans*, in particular the high dose group relative to quercetin, prevented weight loss, likely through the prevention of the breakdown of fats and proteins for glucose production. The process of gluconeogenesis is potentiated in T2D rats (Imam & Maznah, 2013) to provide additional supply of glucose because the cells of the body do not uptake enough for the metabolism. The substrates for gluconeogenesis are derived from protein and fat sources, potentially reducing these body stores and causing weight loss (Fine & Feinman, 2004). If gluconeogenesis was attenuated, the extract would have slowed down the breakdown of body fat and proteins thereby preventing the weight loss associated with it. No differences in serum insulin levels between the diabetic control and the treatment groups suggest that the differences observed in glycemia may have been due to the enhanced effects of the extracts on insulin levels or an insulin‐like action.

### 3.3 Lipid profile, liver enzymes, creatinine, and urea parameters

Lipid profiles, liver enzymes (alanine transaminase, aspartate transaminase, and alkaline phosphatase), creatinine, and urea were worse in the diabetic groups in comparison with the normal group at the beginning of the intervention (*p < *0.05) (Table 2). At the end of the intervention, the extracts showed less signs of toxicity to the liver and kidney as indicated by the liver enzymes, serum creatinine, and urea values. However, these values were not statistically lower than those of the diabetic control group except for alkaline phosphatase, which was lower in the *C. nutans* groups. While for cholesterol, at 4 weeks of intervention, the level was significantly lower in the *C. nutans* groups in comparison with the diabetic control group (*p < *0.05); the levels of cholesterol in these groups were comparable to the normal and quercetin‐treated groups. Similar results were observed for low density lipoprotein (LDL) cholesterol, while high density lipoprotein (HDL) cholesterol was elevated by the extracts to levels that were comparable to the normal and quercetin groups (*p < *0.05). Furthermore, triglycerides were reduced in both extract groups to a greater extent than in the quercetin group (*p < *0.05).

Dyslipidemia in T2D is a major cause of cardiovascular disease. The management of T2D must therefore consider lipid abnormalities to prevent cardiovascular complications and the resulting consequences (Palazhy & Viswanathan, 2017). Additionally, the liver and kidneys metabolize exogenous compounds and remove them from the body. These organs are also targets for insults in T2D rats, and their altered functions can be detected using liver enzymes, serum urea, and creatinine (Imam, Musa, Azmi, & Ismail, 2012). In fact, damage to these organs is the basis for some life‐threatening complications of T2D. It is therefore an important goal in managing T2D to not only improve glycemic control but to also prevent damage to these vital organs. Overall, T2D‐induced lipid profile abnormalities and toxicities to the liver and kidneys were attenuated by *C. nutans *aqueous extract. The data suggest that aqueous leaf extract of *C. nutans* may be effective at reducing total cholesterol, improving glycemia, attenuating T2D‐induced dyslipidemia, liver and kidney perturbations, thus resulting in a reduced risk of diabetic complications.

### 3.4 Kidney, lens, and sciatic nerve sorbitol contents and activities of aldose reductase and sorbitol dehydrogenase

**Figure 2:**
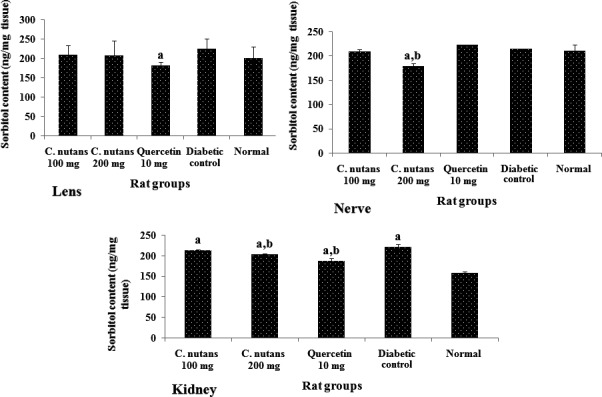
Sorbitol contents of the lens, nerve, and kidney at 4 weeks of intervention in T2D rats. Groups are the same as in Table 1. ^a^indicates a statistical difference in comparison with the normal group for either glucose or weight measurements in each column (*p < *0.05). ^b^indicates a statistical difference in comparison with the diabetic control group for either glucose or weight measurements in each column (*p < *0.05)

Analyses of the sorbitol contents (Figure 2) of the kidney, lens, and nerve revealed that it was elevated in diabetes, although more markedly so in the kidneys. Quercetin, which is known to lower sorbitol levels (Kador, Kinoshita, & Sharpless, 1985), reduced sorbitol significantly in the lens and kidneys but not in the nerve. The aqueous extracts also reduced the sorbitol contents in the organs, but only those of the kidney and nerve were significantly reduced in comparison with the diabetic control group (*p < *0.05).

**Figure 3:**
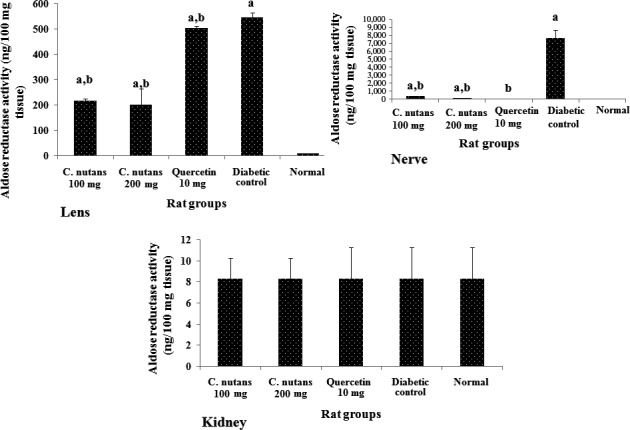
Aldose reductase activities in the lens, nerve, and kidney at 4 weeks of intervention in T2D rats. Groups are the same as in Table 1. ^a^indicates a statistical difference in comparison with the normal group for either glucose or weight measurements in each column (*p < *0.05). ^b^indicates statistical difference in comparison with the diabetic control group for either glucose or weight measurements in each column (*p < *0.05)

The aldose reductase activity (Figure 3) was significantly higher in the lens and nerve, but not in kidney of diabetic control group in comparison with the normal group. Additionally, quercetin and the aqueous extracts reduced aldose reductase activities in the lens and nerve, although the extracts showed better results in the lens, while the results were comparable to those of quercetin group for the nerve. No significant differences (*p < *0.05) were observed in kidney aldose reductase activity. The reduced aldose reductase activity in the lens and nerve likely contributed to the lower sorbitol contents of the lens and nerve (Yabe‐Nishimura, 1998), but the activity of this enzyme in the kidney cannot be said to account for the differences in the sorbitol content of the kidney. The metabolic flux due to high blood glucose is a contributing factor to the level of sorbitol deposited in tissues in diabetes. Hence, better glycemic control following treatment with the extracts could have led to lower sorbitol contents in the kidneys due to less substrate (glucose) for sorbitol production (Gabbay, 1975). Moreover, sorbitol dehydrogenase activity may also determine levels of sorbitol since it can breakdown sorbitol to fructose (Kador et al., 1985), which is then utilized for energy.

**Figure 4:**
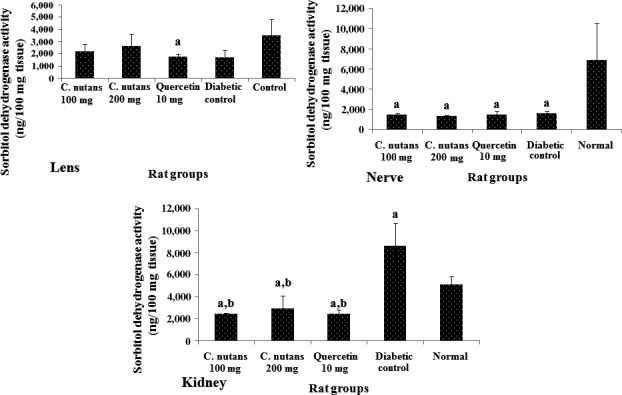
Sorbitol dehydrogenase activities in the lens, nerve, and kidney at 4 weeks of intervention in T2D rats. Groups are the same as in Table 1. ^a^indicates a statistical difference in comparison with the normal group for either glucose or weight measurements in each column (*p < *0.05). ^b^indicates a statistical difference in comparison with the diabetic control group for either glucose or weight measurements in each column (*p < *0.05)

Sorbitol dehydrogenase activity (Figure 4) was higher in the lens of the *C. nutans* aqueous leaf extract‐treated rats in comparison with other diabetic rats, while the activity of the enzyme was similar in the nerve and kidneys of the diabetic groups, except in the kidneys where the diabetic control group showed an unexpectedly high level of activity. This may be a counter‐mechanism by which the kidneys regulated the sorbitol level in the diabetic control group. This may also be the basis for the similar sorbitol levels in the kidneys of the diabetic control group and other groups. The ability of *C. nutans* aqueous leaf extract to regulate the buildup of sorbitol, especially in the kidney and nerve, suggests that it could be used to manage diabetic neuropathy and nephropathy due to sorbitol accumulation in these organs (Matough, Budin, Hamid, Alwahaibi, & Mohamed, 2012).

### 3.5 Oxidative stress markers

**Figure 5:**
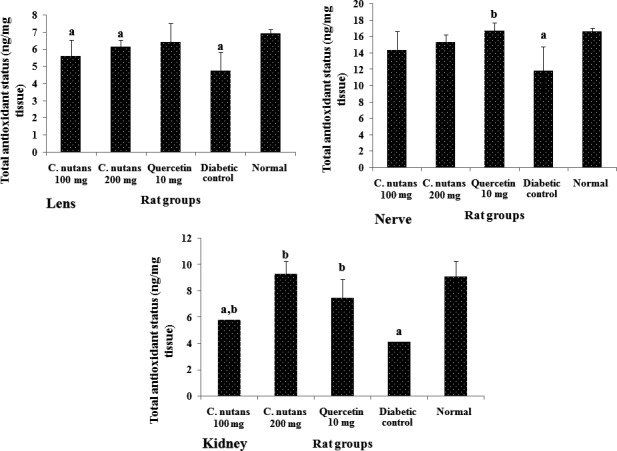
Total antioxidant status of the lens, nerve, liver, and kidney at 4 weeks of intervention in T2D rats. Groups are the same as in Table 1. ^a^indicates a statistical difference in comparison with the normal group for either glucose or weight measurements in each column (*p < *0.05). ^b^indicates a statistical difference in comparison with the diabetic control group for either glucose or weight measurements in each column (*p < *0.05)

**Figure 6:**
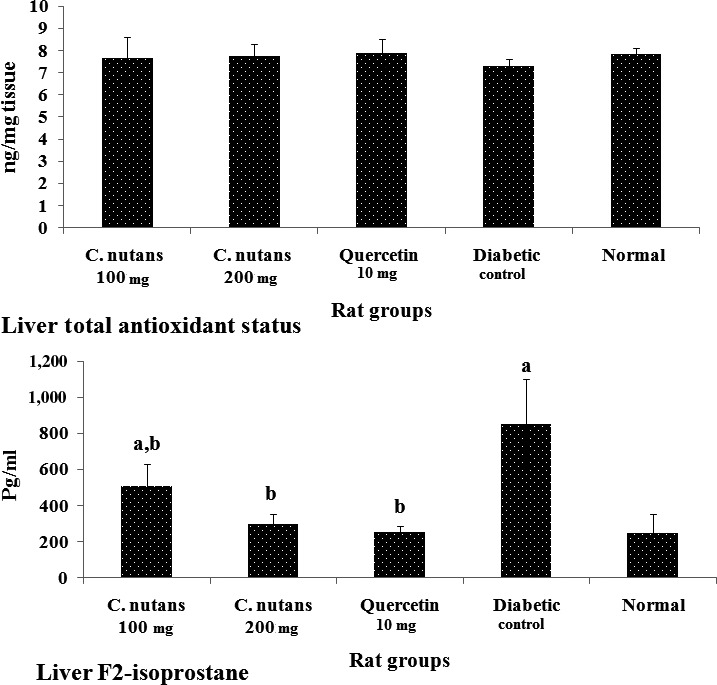
Liver total antioxidant status (6a) and F2‐isoprostane levels (6b) at 4 weeks of intervention in T2D rats. Groups are the same as in Table 1. ^a^indicates a statistical difference in comparison with the normal group for either glucose or weight measurements in each column (*p < *0.05). ^b^indicates a statistical difference in comparison with the diabetic control group for either glucose or weight measurements in each column (*p < *0.05)

The total antioxidant status of the lens, nerve, and kidney is shown in Figure 5, while that of the liver is shown on Figure 6a. The data show that the diabetic control group had lower antioxidant status than the normal group in all organs suggesting that there was a depletion of antioxidant stores due to diabetes mellitus. Additionally, the treatment groups showed better antioxidant status in comparison with the diabetic control group, which indicated that the extracts and quercetin boosted antioxidant levels in the diabetic rats, which may have contributed to the better metabolic outcomes observed in the treated groups. Moreover, improved antioxidant status is associated with better outcomes in T2D (Rahimi, Nikfar, Larijani, & Abdollahi, 2005), and tends to correlate with improved glycemic control (Dey & Lakshmanan, 2013), as seen in this study.

Figure 6b shows liver F2‐isoprostane levels at 4 weeks of intervention. The level of F2‐isoprostane in the diabetic control group was significantly higher than the normal group (*p < *0.05), while the *C. nutans *groups showed attenuated levels. The F2‐isoprostane is a biologically relevant marker of oxidative stress and has been linked to chronic diseases. In T2D, its elevation is linked to worsening of the disease, while reduced levels signal improvement in metabolic indices (Laight, Desai, Gopaul, Anggård, & Carrier, 1999). The significantly higher level of F2‐isoprostane in the diabetic control group compared to the normal (*p < *0.05) and the *C. nutans *groups suggests an improved antioxidant status, in keeping with the total antioxidant status results.

There is evidence to suggest that oxidative stress is elevated in T2D mellitus with corresponding depletion of antioxidant proteins. This situation promotes damage to organs through diabetic complications. Thus, interventions that could boost antioxidant status and/or reduce oxidative stress have been shown to improve metabolic outcomes in T2D mellitus (Maritim, Sanders, & Watkins, 2003; Matough et al., 2012). The attenuation of T2D induced oxidative stress (F2‐isoprostane) and the potentiating of antioxidant status suggests that *C. nutans* can improve metabolic outcomes in T2D through different mechanisms.

### 3.6 Histological changes

**Figure 7:**
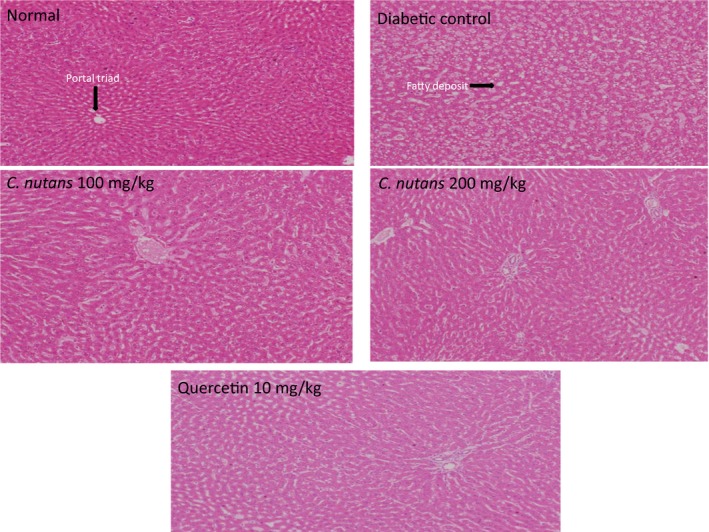
Liver histology at 4 weeks of intervention in T2D rats. 10x Magnification. Groups are the same as in Table 1

**Figure 8:**
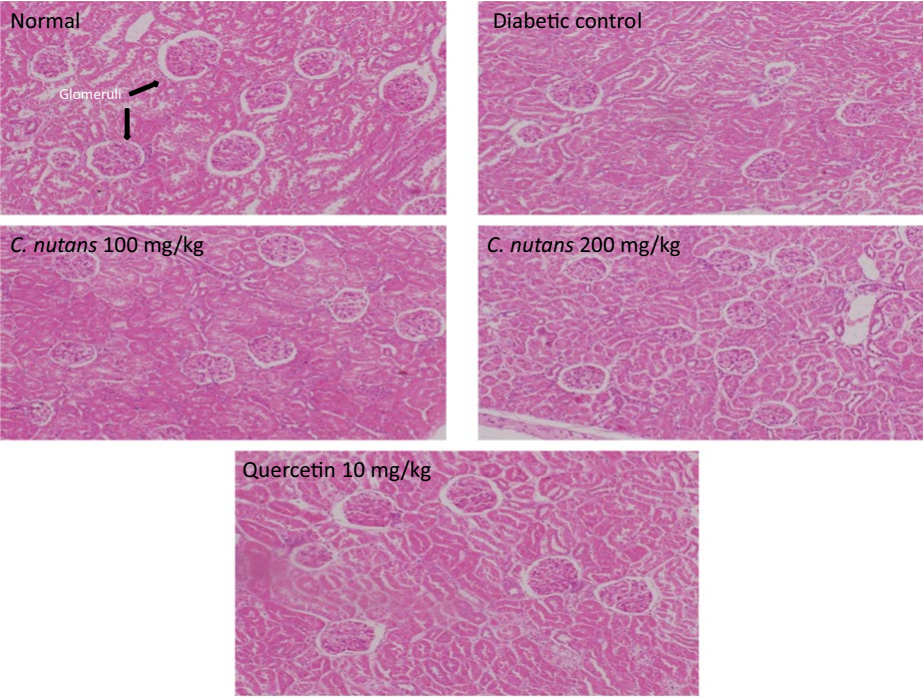
Kidney histology at 4 weeks of intervention in T2D rats. 10x Magnification. Groups are the same as in Table 1

Liver histology showed that untreated diabetes resulted in distortion of the liver architecture, while other groups administered with *C. nutans *extracts and quercetin preserved the architecture to varying degrees (Figure 7). Additionally, the kidney histology also showed fewer glomeruli and distortion of the normal kidney architecture in the diabetic control group, while other treated groups maintained the architecture and glomeruli to varying degrees (Figure 8). The histology results are in agreement with the toxicity data, which suggested preservation of liver and kidney function in the *C. nutans* ‐treated groups in comparison with the diabetic control group. Concerns over the side effects and other adverse effects resulting from synthetic compounds in the treatment of T2D have been factors leading to medicinal plants as alternative choices (Imam et al., 2016). In view of the ability of *C. nutans* aqueous leaf extract to control glycemia, oxidative stress, lipid abnormalities, and sorbitol‐related complications, accompanied with protection of liver and kidney function, it is therefore a good candidate for the management of T2D (Kaur, 2014; Matough et al., 2012; Yabe‐Nishimura, 1998).

## 4 CONCLUSION

In this study, T2D rats treated with an aqueous leaf extract of *C. nutans *had improved glycemic control than those treated with quercetin, while their lipid profiles were significantly improved and comparable to those of the quercetin group. Overall, the data show that *C. nutans *extracts improved glycemic control and complications related to sorbitol accumulation, similar to quercetin, in fact at higher doses (200 mg/kg), *C. nutans* showed better results than quercetin. The total antioxidant capacities of liver, kidney, nerve, and lens in treated rats were also comparable to the quercetin treatment. Sorbitol levels, aldose reductase, and sorbitol dehydrogenase activities in the kidney, lens, and sciatic nerve were improved by the plant extract to levels that were comparable to those of the quercetin group. No signs of toxicity were seen in the kidneys or liver. The *C. nutans* has been used traditionally to manage metabolic diseases in parts of Asia, and the present results provide evidence for the efficacy of the plant. The use of an aqueous extract in this study further corroborates the efficacies observed in its traditional. Hence, *C. nutans *has medicinal properties that can be developed into nutraceuticals for the management of diabetes and its complications and other related metabolic disorders with lipid abnormalities and oxidative stress. However, clinical and long‐term studies are indicated to confirm the translational implications of these findings.

## ETHICAL APPROVAL

All experimental protocols and procedures were reviewed and approved by Animal Care and Use Committee (ACUC) of the Faculty of Medicine and Health Sciences, University Putra Malaysia (Project approval number: UPM/IACUC/UAP‐R045/2013).

## CONFLICT OF INTEREST

Annie George and Sasikala Chinnappan are employees of Biotropics Malaysia Berhad who funded the research. Dr. Mustapha Umar Imam, Prof. Maznah Ismail, and Dr. Ashril Yusof declare no conflict of interest.

## DATA AVAILABILITY

The authors confirm that the data supporting the findings of this study are available within the article.
